# Red blood cell transfusion in neurocritical patients: a systematic review and meta-analysis

**DOI:** 10.1186/s12871-024-02487-9

**Published:** 2024-03-19

**Authors:** Yun Yu, Yuxuan Fu, Wenying Li, Tiantian Sun, Chan Cheng, Yingzi Chong, Ruquan Han, Weihua Cui

**Affiliations:** 1 Department of Anesthesiology, Beijing Tiantan Hospital, Capital Medical University, No. 119, Southwest 4th Ring Road, Fengtai District, Beijing, 100070 PR China; 2 Department of Anesthesiology, Tsinghua University Yuquan Hospital, 5 Shijingshan Rd, Shijingshan District, Beijing, PR China; 3 Department of Anesthesiology, Beijing Anzhen Hospital, Capital Medical University, 2 Anzhen Road, Beijing, PR China; 4 Department of Anesthesiology, Beijing Stomatological Hospital Affiliated to Capital Medical University, No.4 Tiantan Xili, Dongcheng District, Beijing, 100050 PR China; 5 Department of Anaesthesiology, First Affiliated Hospital of Soochow University, Suzhou, Jiangsu PR China

**Keywords:** Red blood cell transfusion, Neurocritical, Meta-analysis, Systematic review

## Abstract

**Background:**

Anemia can lead to secondary brain damage by reducing arterial oxygen content and brain oxygen supply. Patients with acute brain injury have impaired self-regulation. Brain hypoxia may also occur even in mild anemia. Red blood cell (RBC) transfusion is associated with increased postoperative complications, poor neurological recovery, and mortality in critically ill neurologic patients. Balancing the risks of anemia and red blood cell transfusion-associated adverse effects is challenging in neurocritical settings.

**Methods:**

We searched the Cochrane Central Register of Controlled Trials (CENTRAL), Embase, and MEDLINE (PubMed) from inception to January 31, 2024. We included all randomized controlled trials (RCTs) assessing liberal versus restrictive RBC transfusion strategies in neurocritical patients. We included all relevant studies published in English. The primary outcome was mortality at intensive care unit (ICU), discharge, and six months.

**Results:**

Of 5195 records retrieved, 84 full-text articles were reviewed, and five eligible studies were included. There was no significant difference between the restrictive and liberal transfusion groups in ICU mortality (RR: 2.53, 95% CI: 0.53 to 12.13), in-hospital mortality (RR: 2.34, 95% CI: 0.50 to 11.00), mortality at six months (RR: 1.42, 95% CI: 0.42 to 4.78) and long-term mortality (RR: 1.22, 95% CI: 0.64 to 2.33). The occurrence of neurological adverse events and most major non-neurological complications was similar in the two groups. The incidence of deep venous thrombosis was lower in the restrictive strategy group (RR: 0.41, 95% CI: 0.18 to 0.91).

**Conclusions:**

Due to the small sample size of current studies, the evidence is insufficiently robust to confirm definitive conclusions for neurocritical patients. Therefore, further investigation is encouraged to define appropriate RBC transfusion thresholds in the neurocritical setting.

**Supplementary Information:**

The online version contains supplementary material available at 10.1186/s12871-024-02487-9.

## Introduction

Common neurocritical diseases include subarachnoid hemorrhage (SAH), traumatic brain injury (TBI), and intracerebral hemorrhage (ICH). Most SAH is caused by the rupture of intracranial aneurysms [1]. Aneurysmal SAH occurs in 2–16 of every 100,000 people worldwide, and the mortality remains as high as 32–67% [2]. Anemia occurs in 50% of patients with SAH [3]. Patients undergoing surgical treatment for SAH are at high risk for anemia, with 36% to 47% of patients having hemoglobin below 10 g/dl in the first four days after surgery [4]. More than 10 million people worldwide experience TBI each year [5]. Most patients with TBI also have anemia, and more than one-third receive red blood cell (RBC) transfusions in the intensive care unit (ICU) [6]. Hypertensive ICH is one of the most severe complications of hypertension, with an incidence of 9–28% in Europe and the United States and 19–48% in China [7].

The human brain is one of the most oxygen-efficient organs, so it is highly susceptible to hypoxic conditions [8]. Anemia can lead to secondary brain damage by reducing arterial oxygen content and brain oxygen supply. Although patients with anemia may compensate by increasing the rate of oxygen intake, patients with acute brain injury have impaired reserve. Therefore, brain hypoxia may also occur even in mild anemia [9, 10]. Anemia is an independent risk factor for poor prognosis in neurocritical settings [11]. Previous studies have shown that in patients with TBI, SAH, and ICH, hemoglobin less than 9 g/dl is associated with tissue hypoxia and poor prognosis [11]. However, RBC transfusion is associated with increased postoperative complications, poor neurological recovery, and mortality in critically ill neurologic patients [11–15]. Most of the current evidence is based on association studies with no causality. Therefore, we need to cautiously formulate blood transfusion indications suitable for these patients according to their characteristics.

Current guidelines recommend restricted RBC transfusion strategies (hemoglobin transfusion threshold of 7 g/dL) for critical patients with hemodynamic stability, except for those with myocardial ischemia [16]. It has also been expressed in guidelines that the existing evidence is insufficient to explain the safety of RBC transfusion strategies in neurocritical settings [17–20]. Therefore, we conducted an updated systematic review and meta-analysis to explore RBC transfusion strategies for neurocritical patients.

## Materials and methods

### Protocol and registration

We followed the principle of Preferred Reporting Items for Systematic Reviews and Meta-Analyses (PRISMA). The registration number of this review was CRD42021225043 on the International Prospective Register of Systematic Reviews (PROSPERO).

### Search methods for the identification of studies

We searched the Cochrane Central Register of Controlled Trials (CENTRAL), Embase, and MEDLINE (PubMed). YY and YF performed the search on 31 January 2024. We used sensitivity- and precision-maximizing search terms described in the Cochrane Handbook to search for eligible studies in Embase and PubMed [21]. The search strategies were described in the Supplemental Digital Content.

We reviewed the references in all eligible articles and reviews to identify further studies. For ongoing studies, we searched ClinicalTrials.gov on 3 February 2024. We imposed no regional restrictions.

### Selection criteria

We included all randomized controlled trials (RCTs) assessing liberal versus restrictive RBC transfusion strategies in neurocritical patients, including TBI, ICH, and SAH. We included all relevant studies published in English regardless of publication status. We excluded nonrandomized studies, such as cohort studies, which are susceptible to bias. We included adult participants (aged 18 years or older) with TBI, ICH, and aneurysmal SAH, either undergoing surgery or not. We excluded those participants with severe extracranial trauma.

A liberal strategy for RBC transfusion was used in the intervention group. A restrictive strategy for RBC transfusion was applied in the control group. The authors in each included study defined the RBC transfusion threshold. Primary outcomes included mortality at ICU, discharge, and six months. Secondary outcomes included unfavorable Glasgow Outcome Score (GOS) at six months; neurological adverse events; any major non-neurological complications; adverse events; ICU and hospital lengths of stay. Neurological adverse events included vasospasm, stroke and intracranial hypertension requiring therapy. Vasospasm was defined as a middle cerebral velocity greater than 120 cm/sec in one vascular territory associated with a Lindegaard Index greater than 3 [22]. The treatment of intracranial hypertension included the administration of hyperosmolar agents, vasopressors, the use of hyperventilation, sedation, analgesia, neuromuscular blockers, cerebral spinal fluid drainage, diuretics, hypothermia, barbiturates or decompressive craniectomy [22]. Major non-neurological complications included deep venous thrombosis (DVT), acute myocardial infarction, hypotension, pneumonia, pulmonary embolus, acute respiratory distress syndrome and urinary tract infection.

### Data collection and analysis

Two review authors (YY and YF) independently performed the search. Two review authors (YY and WL) independently screened the search results and separately collected the reasons for inclusion or exclusion. Two review authors (YF and TS) extracted data independently using an electronic data extraction form. Two review authors discussed and resolved disagreements. If necessary, we consulted with a third review author (WC or RH). Two review authors (TS and YC) analyzed data independently. YY contacted the corresponding authors by e-mail if further information was needed.

Two review authors (YY and CC) independently evaluated the risk of bias in the included studies. We used the risk-of-bias tool described in the Cochrane Handbook for Systematic Reviews of Interventions [21]. We appraised each study according to the following domains: selection bias, performance bias, and attrition bias, including randomization and allocation concealment, blinding of participants, blinding of outcome assessment, missing data, selective reporting, and any other bias. If all domains were identified as adequate, the study was rated as having a low risk of bias. If one or more domains were assessed as inadequate or unclear, the study was rated as having a high risk of bias. A risk-of-bias graph with the categories of low, high, or unclear risk of bias was made to demonstrate review authors’ judgments about each risk of bias item across all included studies.

We performed data analyses using Review Manager software (RevMan 5.4). For dichotomous data, we used a random effects model to analyze the overall risk ratio (RR) with a 95% confidence interval (CI). For continuous data, mean differences (MDs) were used as summary statistics in the data analysis. We used Review Manager software to pool the data on participants, interventions, and outcomes and generated a quantitative summary by performing a meta-analysis. Clinical and methodological heterogeneity was expected among studies. Consequently, we analyzed the data using a random-effects model. If the numerical data were insufficient for a meta-analysis, we conducted a narrative analysis for each eligible study.

We evaluated the heterogeneity based on clinical diversity (e.g., type of cerebral hemorrhage, different RBC transfusion thresholds) and methodological diversity. If necessary, we conducted subgroup or sensitivity analysis to address clinical heterogeneity, including visual observation of the forest plots and the I^2^ statistic [21]. An I^2^ statistic of over 50% indicated high levels of heterogeneity, which mandated further analysis. Only five studies were involved in the meta-analysis. Therefore, we did not make a funnel plot to appraise publication or reporting bias qualitatively. As the included studies were insufficient, we did not perform subgroup analysis according to different diseases, including TBI, ICH, and SAH.

### “Summary of findings” table and GRADE

We used the principles of the GRADE system to assess the quality of the body of evidence associated with the primary outcomes. We constructed a “Summary of findings” table using the GRADEpro software [23]. We generated a “Summary of findings” table for “mortality for neurocritical patients” (Supplemental Table 1).

## Results

### Study selection

**Fig. 1:**
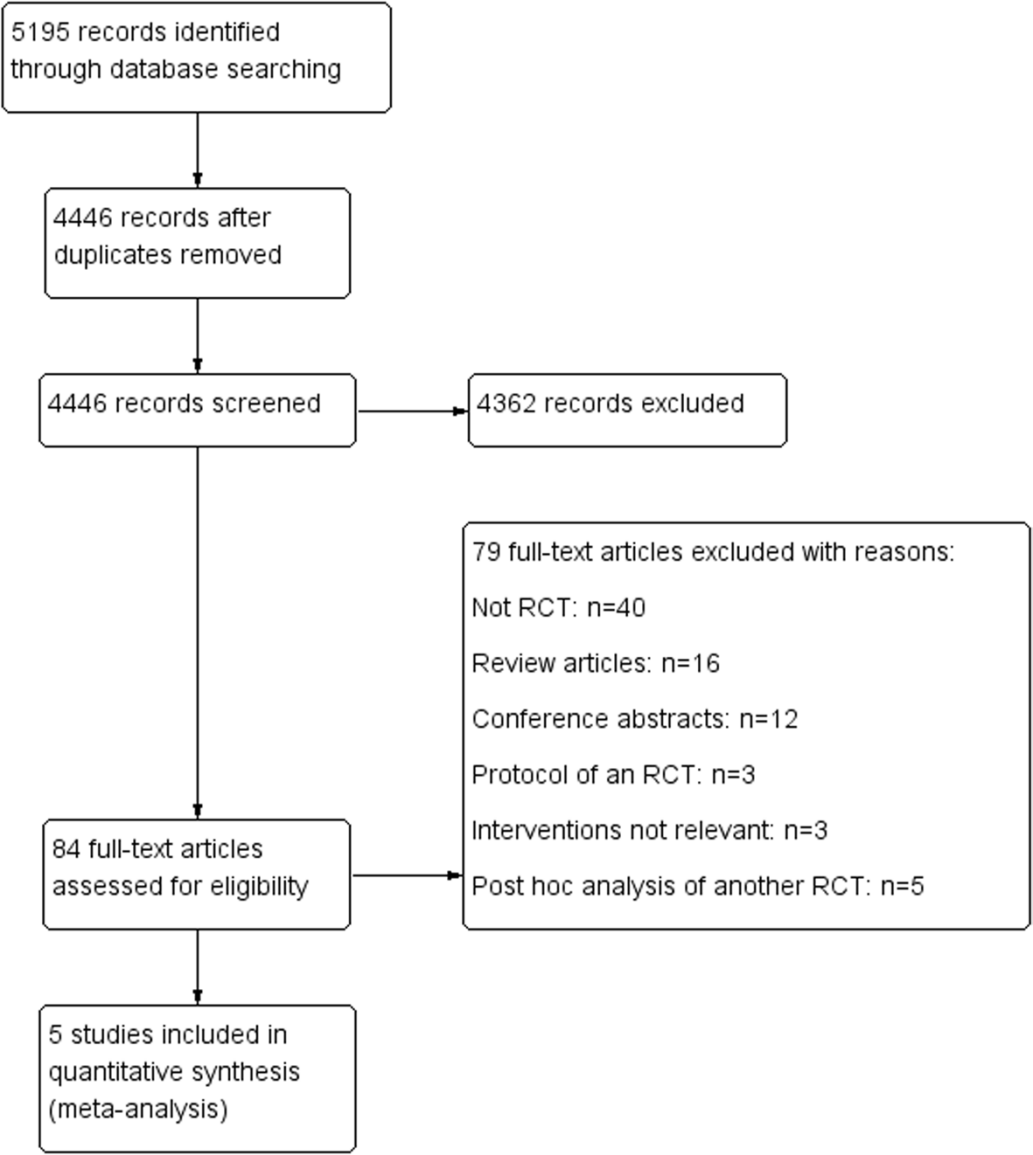
Flowchart. RCT, randomized controlled trial

There were 5195 bibliographic citations identified after we conducted a systematic search for the information following the strategy. After 749 duplicates were excluded, our database search identified 4446 potentially relevant records. Five studies were considered eligible based on the titles, abstracts, and full texts. A flowchart for identification is shown in Fig. 1.

### Characteristics of the included studies

The five included RCTs were published between 2006 and 2019. Four studies involved patients with TBI [22, 24–26] and one with SAH [27]. The sample size of the included trials ranged from 44 to 200. Supplemental Table 2 provides further clarifications of the included studies.

### Risk of bias and quality assessment

**Fig. 2:**
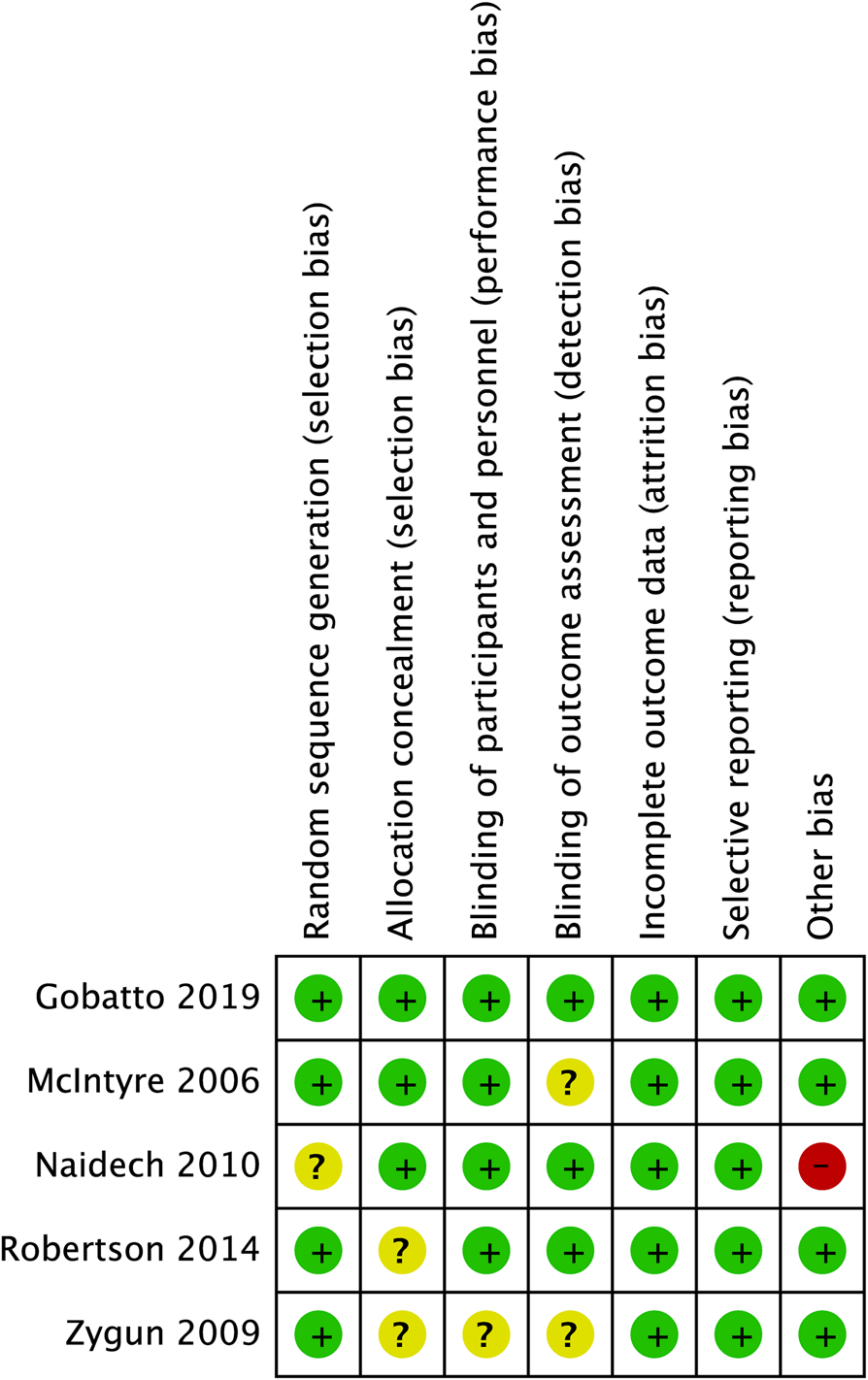
Risk of bias summary

 Four of the five studies included specific procedures for generating random sequences [22, 24–26], and three used sealed envelopes for distribution concealment. Three studies evaluated postoperative adverse events by a blinded researcher [22, 25, 27]. Given the nature of the intervention, the investigator could not be blinded to the treatment strategy. Four studies did not state how they were funded, and it was unclear whether commercial sponsors were involved [22, 24–26]. The other study had no apparent commercial involvement [27]. The risk of bias is summarized in Fig. 2.

### Synthesis of results

Mortality, unfavorable outcomes, neurological complications, major non-neurological complications, and length of ICU or hospital stay with the restrictive strategy and the liberal strategy were evaluated in this review.

### Mortality at ICU, discharge, six months and long-term mortality

**Fig. 3:**
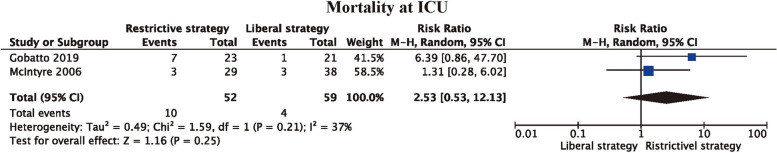
The impact of different transfusion strategies on mortality at ICU. ICU, Intensive care unit

**Fig. 4:**
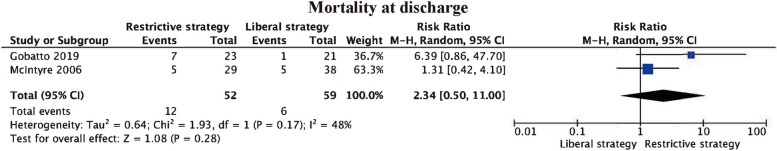
The impact of different transfusion strategies on mortality at discharge

**Fig. 5:**
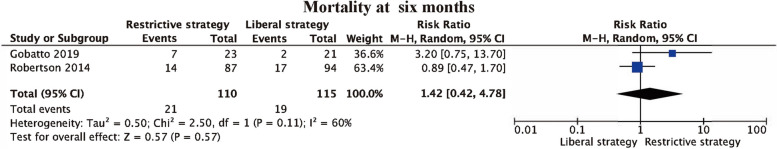
The impact of different transfusion strategies on mortality at six months

**Fig. 6:**
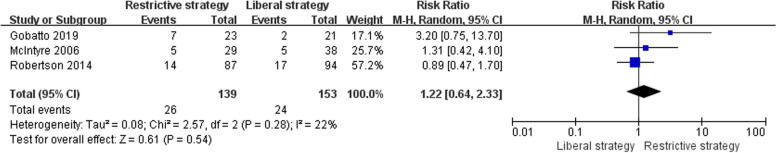
The impact of different transfusion strategies on long-term mortality

In two of the five studies, mortality during the ICU stay was assessed (Fig. 3) [22, 26]. Both studies showed no significant difference in mortality during the ICU stay between the restrictive and liberal transfusion groups. The combined results of both studies revealed no significant difference in ICU mortality between the two groups with low heterogeneity (19.2% for restrictive strategy vs. 6.8% for liberal strategy, RR: 2.53, 95% CI: 0.53 to 12.13, I^2^ = 37%). In two studies that included 111 patients, mortality at discharge was assessed (Fig. 4) [22, 26]. Both studies showed no significant difference in in-hospital mortality between the restrictive and liberal transfusion groups. Overall, the combined results of both studies revealed no significant difference in in-hospital mortality between the two groups (23.1% for restrictive strategy vs. 10.2% for liberal strategy, RR: 2.34, 95% CI: 0.50 to 11.00, I^2^ = 48%). In two of the five studies, mortality at six months was assessed (Fig. 5) [22, 25]. The combined results of both studies revealed that there was no significant difference in 6-month mortality between the two groups with substantial heterogeneity (19.1% for restrictive strategy vs. 16.5% for liberal strategy, RR: 1.42, 95% CI: 0.42 to 4.78, I^2^ = 60%). Mortality at 60 days and mortality at 6 months were pooled as long-term mortality, including 292 patients. There was no significant difference in long-term mortality between the two groups (18.7% for restrictive strategy vs. 15.7% for liberal strategy, RR: 1.22, 95% CI: 0.64 to 2.33, I^2^ = 22%) (Fig. 6) [22, 25, 26].

### Unfavorable outcomes

Data on unfavorable GOS outcomes at six months were obtained from two trials (Supplemental Figure 1) [22, 25], including 225 patients, and no clear evidence of differences was seen between the two groups (57.3% for restrictive strategy vs. 61.7% for liberal strategy, RR: 1.03, 95% CI: 0.62 to 1.73, I^2^ = 59%). Independence at three months was reported from one study and dependence at three months was calculated [27]. Dependence at 3 months and unfavorable GOS outcomes at six months were pooled as long-term unfavorable outcomes, including 269 patients [22, 25, 27]. The combined results showed no significant difference between the two groups (49.6% for restrictive strategy vs. 54.4% for liberal strategy, RR: 0.96, 95% CI: 0.69 to 1.34, I^2^ = 19%) (Supplemental Figure 2).

### Patients transfused

Three studies reported the number of patients who received red blood cell transfusions (Supplemental Figure 3) [22, 25, 27]. The overall incidence was 57.9% in the restrictive strategy group and 79.7% in the liberal strategy group (RR: 0.74, 95% CI: 0.59 to 0.92, I^2^ = 55%). In two of the five studies, the red blood cell units per patient were assessed (Supplemental Figure 4) [22, 26]. The combined results showed that red blood cell units per patient in the restrictive strategy group were significantly lower than those in the liberal group (MD: -2.37, 95% CI: -3.94 to -0.81, I^2^ = 77%).

### Neurological adverse events

#### Vasospasm

Two studies reported this outcome (Supplemental Figure 5) [22, 27]. Overall, the results showed no significant difference in the incidence of cerebral vasospasm between the two groups (RR: 1.82, 95% CI: 0.50 to 6.67, I^2^ = 69%).

#### Stroke

Stroke incidence has been reported in three studies (Supplemental Figure 6) [22, 25, 27]. The combined results showed no significant difference in the incidence of stroke between the two groups (RR: 0.99, 95% CI: 0.40 to 2.48, I^2^ = 9%).

#### Intracranial hypertension requiring therapy

The incidence of increased intracranial hypertension requiring treatment was assessed in two of the five studies (Supplemental Figure 7) [22, 25]. There was no significant difference in the incidence between the two groups (RR: 1.01, 95% CI: 0.82 to 1.25, I^2^ = 41%). However, the incidence of cranial hypertension varied widely across all studies, ranging from 100% to 39.4%.

### Major nonneurological complications

The incidence of DVT was reported in two studies involving 244 participants (Supplemental Figure 8) [22, 25]. The values of overall incidence were 5.7% and 15.6% in the restrictive strategy group and the liberal strategy group, respectively (RR: 0.41, 95% CI: 0.18 to 0.91, I^2^ = 0%). There was no significant difference between the two groups in acute myocardial infarction (Supplemental Figure 9) (RR: 1.02, 95% CI: 0.06 to 16.09), hypotension (Supplemental Figure 10) (RR: 1.05, 95% CI: 0.85 to 1.29, I^2^ = 0%), pneumonia (Supplemental Figure 11) (RR: 0.93, 95% CI: 0.50 to 1.72, I^2^ = 21%), pulmonary embolus (Supplemental Figure 12) (RR: 0.32, 95% CI: 0.06 to 1.68, I^2^ = 0%), acute respiratory distress syndrome (ARDS) (Supplemental Figure 13) (RR: 1.05, 95% CI: 0.40 to 2.76, I^2^ = 48%), and urinary tract infection (Supplemental Figure 14) (RR: 0.60, 95% CI: 0.08 to 4.65, I^2^ = 51%).

### ICU and hospital lengths of stay

Two studies involving 111 participants provided ICU length of stay and hospital length of stay as skewed data [22, 26]. Gobatto 2019 [22] and McIntyre 2006 [26] demonstrated no difference between the restrictive strategy group and the liberal strategy group in the length of ICU stay (in days) (16 (13–18) versus 21 (9–30) and 10 (5–21) versus 8 (5–11), respectively). Additionally, there was no difference in the length of hospital stay (in days) (42 (23–76) versus 35 (21–63) and 27 (14, 39) versus 30.5 (17, 47), respectively).

### Ongoing studies

By searching the clinical trial registry platforms, we identified three ongoing trials without available results from the investigators. Supplemental Table 3 provides the characteristics of the ongoing trials. One of the ongoing studies was conducted in a TBI setting (HEMOTION, https://clinicaltrials.gov/ct2/show/record/NCT03260478), one study in an SAH setting (SAHaRA, https://clinicaltrials.gov/ct2/show/record/NCT03309579), and one study in an acute brain injury setting (TRAIN, https://clinicaltrials.gov/ct2/show/NCT02968654). Three of these studies recruited participants. Protocols of these ongoing studies have been published [28–30].

### Summary of findings

Using the principles of the GRADE system, we found a low-quality body of evidence associated with ICU mortality and in-hospital mortality. We found a very low-quality body of evidence associated with mortality at 6 months (Supplemental Table 1).

## Discussion

The principal finding of our meta-analysis was that there was no significant difference between the restrictive strategy and liberal strategy in mortality, unfavorable outcomes, neurological adverse events, and most major non-neurological complications. Fewer patients received RBC transfusions in the restrictive strategy group. In addition, the incidence of DVT was lower in the restrictive strategy group. However, currently available data is limited. It remains uncertain which transfusion strategy is more appropriate for neurocritical patients.

### Causes of anemia in neurocritical patients

Many factors can lead to anemia in critically ill patients. In addition to direct blood loss, including venous blood dilution caused by fluid resuscitation, inflammatory cytokines cause a secondary decrease in erythropoietin and a change in red bone marrow precursor cell proliferation and differentiation [31]. Abnormal metabolism of folic acid, vitamin B12, iron, and other nutrients can lead to reduced erythropoiesis [32]. In severe patients, the systemic inflammatory reaction can shorten the lifespan of red blood cells, and oxidative stress can directly induce RBC apoptosis [32].

### Effects of anemia on the central nervous system

Tissue oxygen delivery depends on organ blood flow and arterial oxygen content, which is related to hemoglobin concentration and oxygen saturation [33]. The oxygen supply of the central nervous system depends on several variables. Cerebral oxygen availability is the product of cerebral blood flow (CBF) and arterial oxygen content [11]. Therefore, increasing local blood flow and oxygen uptake rate to maintain adequate oxygen is necessary during anemia. Under healthy conditions, the oxygen uptake rate of brain tissue remains high, which limits its ability to compensate by increasing the rate of oxygen uptake in anemia [12]. Therefore, the brain compensates mainly by dilating cerebral vessels and increasing CBF [12]. In healthy volunteers, when hemoglobin drops to approximately 5 g/dL, the brain cannot compensate for inadequate oxygen delivery by further increasing CBF [12, 34]. The end expiratory partial pressure of carbon dioxide has an important effect on cerebral blood flow regulation [35]. This phenomenon is called cerebral carbon dioxide reaction and reflects the capacity of cerebral blood vessels to reserve [35]. A prospective study with an average follow-up of up to 28 months found a link between impaired cerebrovascular reactivity and cerebral ischemic events [36]. In acute brain injury, cerebrovascular self-regulation function is impaired, cerebrovascular reserve is insufficient, and hypoxia of brain cells may also occur when hemoglobin levels are elevated [12].

### Potential risks of transfusion for critically ill patients

Except for transfusion-related pathogen transmission and infections, the most harmful adverse reactions in neurocritical patients are transfusion-associated circulatory overload (TACO) and transfusion-related acute lung injury (TRALI) [31, 37]. The incidence of TACO and TRALI is 0.06-6% and 5–8% in critically ill patients, respectively [31]. The risk increases with an increasing amount of blood products transfused. Despite different pathophysiological mechanisms, both of these complications lead to increased pulmonary edema, which may decrease survival in critically ill patients [38].

### Controversy over transfusion strategies for neurocritical patients

However, the current research results are still controversial. The clinical studies examining transfusion thresholds in neurocritical patients are weak and results are conflicting [39]. The prospective study found that hemoglobin concentrations less than 9 g/dL were associated with cerebral hypoxia (OR = 7.92; 95% CI, 2.32 to 27.09) and cell dysfunction (OR = 4.24; 95% CI, 1.33 to 13.55) [40]. A retrospective cohort study found that hemoglobin levels lower than 9 g/dl in patients with severe craniocerebral injury were associated with increased mortality (RR = 3.1, 95% CI 1.5–6.3) [39]. Studies have shown that higher hemoglobin levels are associated with improved outcomes in patients with SAH [4, 41, 42]. A RCT found that applying higher target hemoglobin in patients with SAH was safe and feasible [27].

A worrying complication of subarachnoid hemorrhage is cerebral vasospasm [43, 44]. This is the difference between patients with aneurysmal subarachnoid hemorrhage and other neurocritical patients. In patients who rupture for the first time and survive, cerebral vasospasm is the leading cause of death [44]. An observational study found that postoperative RBC transfusion increased the risk of cerebral vasospasm in patients with aneurysmal SAH (OR = 1.68; 95% CI, 1.02 to 2.75) [45]. A cohort study demonstrated that RBC transfusion did not improve the prognosis of patients with SAH after controlling for other confounding factors [3]. Erythrocyte transfusion after TBI was associated with an increase in mortality (RR = 1.23; 95% CI 1.13–1.33) and complications (RR = 1.38; 95% CI 1.32 to 1.44) [46]. Blood transfusion may be associated with other organ injuries, leading to poor prognosis. A RCT found that maintaining a hemoglobin concentration greater than 10 g/dl did not improve 6-month outcomes in patients with TBI. The blood transfusion threshold of 10 g/dl was associated with a higher incidence of thromboembolic events (OR = 0.32; 95% CI 0.12 to 0.79) [25]. A retrospective study found a higher incidence of neurological adverse events among patients with TBI who received RBC transfusion (RR = 3.40; 95% CI 1.35–8.56) and a longer ICU stay (RR = 1.42; 95% CI 1.06 to 1.92) [47]. Moman et al. found that RBC transfusion was associated with longer hospital and ICU length of stays in TBI patients with moderate anemia [48]. A large observational study found that, RBC transfusion may be associated with worsened long-term neurological outcomes in patients with TBI [49]. Of course, this correlation may be due to the fact that patients requiring transfusion therapy are more critically ill. A secondary analysis found that a higher transfusion threshold of 10 g/dl after severe TBI increased the risk of severe progressive hemorrhagic injury events [50].

### Limitations

This review had several limitations. First, quality of the evidence was low or very low due to risk of bias, heterogeneity, and small sample sizes. Second, the definition of restrictive and liberal RBC transfusion threshold varied among studies, which lead to heterogeneity and made the results hard to interpret. We need to wait for the results of the ongoing studies to clarify optimized transfusion threshold. Until then, it is reasonable to use an individualized approach incorporating physiologic and clinical data based on the current guidelines. Third, observational studies were excluded for potential bias although they could provide more real-world information. In addition, patients’ vascular reserve needs to be considered in the heterogeneity assessment, but we do not have access to these data.

## Conclusions

Anemia is associated with poor prognosis and increased mortality in neurocritical patients, and the optimal transfusion threshold is unclear. Due to the small sample size of current studies, the evidence is insufficiently robust to confirm definitive conclusions for neurocritical patients. Therefore, further investigation is encouraged to define appropriate RBC transfusion thresholds in the neurocritical setting.

### Supplementary Information

**Supplementary Material 1.**

## Data Availability

The datasets will be available from the primary investigator (Weihua Cui, Email: weihuacui@ccmu.edu.cn) upon reasonable request after the publication of the study results.

## References

[1] Neifert SN, Chapman EK, Martini ML, Shuman WH, Schupper AJ, Oermann EK, Mocco J, Macdonald RL (2020). Aneurysmal Subarachnoid Hemorrhage: the last decade. Translational Stroke Res.

[2] Sharma D (2020). Perioperative Management of Aneurysmal Subarachnoid Hemorrhage. Anesthesiology.

[3] English SW, Chasse M, Turgeon AF, Lauzier F, Griesdale D, Garland A, Fergusson D, Zarychanski R, van Walraven C, Montroy K (2018). Anemia prevalence and incidence and red blood cell transfusion practices in aneurysmal subarachnoid hemorrhage: results of a multicenter cohort study. Crit Care.

[4] Yee JN, Koht A, McCarthy RJ, Bebawy JF (2017). Factors associated with blood transfusion during intracranial aneurysm surgery. J Clin Anesth.

[5] Hyder AA, Wunderlich CA, Puvanachandra P, Gururaj G, Kobusingye OC (2007). The impact of traumatic brain injuries: a global perspective. NeuroRehabilitation.

[6] Ruel-Laliberte J, Lessard Bonaventure P, Fergusson D, Lacroix J, Zarychanski R, Lauzier F, Tinmouth A, Hebert PC, Green R, Griesdale D (2019). Effect of age of transfused red blood cells on neurologic outcome following traumatic brain injury (ABLE-tbi study): a nested study of the age of blood evaluation (ABLE) trial. Can J Anaesth.

[7] Yang Y, Pan Y, Chen C, Zhao P, Hang C (2022). Clinical significance of multiparameter intracranial pressure monitoring in the prognosis prediction of hypertensive intracerebral hemorrhage. J Clin Med.

[8] Yu Y, Zhang K, Zhang L, Zong H, Meng L, Han R (2018). Cerebral near-infrared spectroscopy (NIRS) for perioperative monitoring of brain oxygenation in children and adults. Cochrane Database Syst Rev.

[9] Gouvea Bogossian E, Rass V, Lindner A, Iaquaniello C, Miroz JP, Cavalcante Dos Santos E, Njimi H, Creteur J, Oddo M, Helbok R (2022). Factors associated with brain tissue oxygenation changes after RBC transfusion in acute brain injury patients. Crit Care Med.

[10] Florez-Perdomo WA, Garcia-Ballestas E, Martinez-Perez R, Agrawal A, Deora H, Joaquim AF, Quinones-Ossa GA, Moscote-Salazar LR. Hemoglobin levels as a transfusion criterion in moderate to severe traumatic brain injury: a systematic review and meta-analysis. Br J Neurosurg. 2023;37(6):1473-9. 10.1080/02688697.2021.1940850 34148446

[11] Schmitt E, Meybohm P, Neef V, Baumgarten P, Bayer A, Choorapoikayil S, Friederich P, Friedrich J, Geisen C, Guresir E (2022). Preoperative anaemia and red blood cell transfusion in patients with aneurysmal subarachnoid and intracerebral haemorrhage - a multicentre subanalysis of the German PBM Network Registry. Acta Neurochir (Wien).

[12] Badenes R, Oddo M, Suarez JI, Antonelli M, Lipman J, Citerio G, Taccone FS (2017). Hemoglobin concentrations and RBC transfusion thresholds in patients with acute brain injury: an international survey. Crit Care.

[13] Kramer AH, Zygun DA, Bleck TP, Dumont AS, Kassell NF, Nathan B (2008). Relationship between hemoglobin concentrations and outcomes across subgroups of patients with Aneurysmal Subarachnoid Hemorrhage. Neurocrit Care.

[14] George ME, Skarda DE, Watts CR, Pham HD, Beilman GJ (2008). Aggressive red blood cell transfusion: no association with improved outcomes for victims of isolated traumatic brain Injury. Neurocrit Care.

[15] Warner MA, O’Keeffe T, Bhavsar P, Shringer R, Moore C, Harper C, Madden CJ, Sarode R, Gentilello LM, Diaz-Arrastia R (2010). Transfusions and long-term functional outcomes in traumatic brain injury. J Neurosurg.

[16] Napolitano LM, Kurek S, Luchette FA, Corwin HL, Barie PS, Tisherman SA, Hebert PC, Anderson GL, Bard MR, Bromberg W (2009). Clinical practice guideline: red blood cell transfusion in adult trauma and critical care*. Crit Care Med.

[17] Leal-Noval SR, Arellano-Orden V, Munoz-Gomez M, Cayuela A, Marin-Caballos A, Rincon-Ferrari MD, Garcia-Alfaro C, Amaya-Villar R, Casado-Mendez M, Dusseck R (2017). Red blood cell transfusion guided by Near Infrared Spectroscopy in Neurocritically Ill patients with moderate or severe Anemia: a Randomized, Controlled Trial. J Neurotrauma.

[18] Carson JL, Guyatt G, Heddle NM, Grossman BJ, Cohn CS, Fung MK, Gernsheimer T, Holcomb JB, Kaplan LJ, Katz LM (2016). Clinical practice guidelines from the AABB: Red Blood Cell Transfusion thresholds and Storage. JAMA.

[19] Carson JL, Stanworth SJ, Roubinian N, Fergusson DA, Triulzi D, Doree C, Hebert PC (2016). Transfusion thresholds and other strategies for guiding allogeneic red blood cell transfusion. Cochrane Database Syst Rev.

[20] Lessard Bonaventure P, Lauzier F, Zarychanski R, Boutin A, Shemilt M, Saxena M, Zolfagari P, Griesdale D, Menon DK, Stanworth S (2019). Red blood cell transfusion in critically ill patients with traumatic brain injury: an international survey of physicians’ attitudes. Can J Anaesth.

[21] Cochrane. Handbook for systematic reviews of interventions version 6.3. http://www.training.cochrane.org/handbook .

[22] Gobatto ALN, Link MA, Solla DJ, Bassi E, Tierno PF, Paiva W, Taccone FS, Malbouisson LM (2019). Transfusion requirements after head trauma: a randomized feasibility controlled trial. Crit Care.

[23] Guyatt GHOA, Kunz R, Vist GE, Falck-Ytter Y, Schünemann HJ, GRADE Working Group. What is "quality of evidence" and why is it important to clinicians? BMJ. 2008;336(7651):995-8. 10.1136/bmj.39490.551019.BE PMC2364804 18456631

[24] Zygun DA, Nortje J, Hutchinson PJ, Timofeev I, Menon DK, Gupta AK (2009). The effect of red blood cell transfusion on cerebral oxygenation and metabolism after severe traumatic brain injury*. Crit Care Med.

[25] Robertson CS, Hannay HJ, Yamal JM, Gopinath S, Goodman JC, Tilley BC, Epo Severe TBITI, Baldwin A, Rivera Lara L, Saucedo-Crespo H (2014). Effect of erythropoietin and transfusion threshold on neurological recovery after traumatic brain injury: a randomized clinical trial. JAMA.

[26] McIntyre LA, Fergusson DA, Hutchison JS, Pagliarello G, Marshall JC, Yetisir E, Hare GM, Hebert PC (2006). Effect of a liberal versus restrictive transfusion strategy on mortality in patients with moderate to severe head injury. Neurocrit Care.

[27] Naidech AM, Shaibani A, Garg RK, Duran IM, Liebling SM, Bassin SL, Bendok BR, Bernstein RA, Batjer HH, Alberts MJ (2010). Prospective, randomized trial of higher goal hemoglobin after subarachnoid hemorrhage. Neurocrit Care.

[28] Turgeon AF, Fergusson DA, Clayton L, Patton MP, Zarychanski R, English S, Docherty A, Walsh T, Griesdale D, Kramer AH (2022). Haemoglobin transfusion threshold in traumatic brain injury optimisation (HEMOTION): a multicentre, randomised, clinical trial protocol. BMJ Open.

[29] English SW, Fergusson D, Chasse M, Turgeon AF, Lauzier F, Griesdale D, Algird A, Kramer A, Tinmouth A, Lum C (2016). Aneurysmal SubArachnoid Hemorrhage-Red Blood Cell Transfusion and Outcome (SAHaRA): a pilot randomised controlled trial protocol. BMJ Open.

[30] Taccone FS, Badenes R, Rynkowski CB, Bouzat P, Caricato A, Kurtz P, Moller K, Diaz MQ, Van Der Jagt M, Videtta W (2023). TRansfusion strategies in acute brain INjured patients (TRAIN): a prospective multicenter randomized interventional trial protocol. Trials.

[31] Hayes MM, Uhl L (2018). To transfuse or not transfuse: an intensive appraisal of red blood cell transfusions in the ICU. Curr Opin Hematol.

[32] Scharte M, Fink MP (2003). Red blood cell physiology in critical illness. Crit Care Med.

[33] Hare GMT, Tsui AKY, Ozawa S, Shander A (2013). Anaemia: can we define haemoglobin thresholds for impaired oxygen homeostasis and suggest new strategies for treatment?. Best Pract Res Clin Anaesthesiol.

[34] Weiskopf RB, Toy P, Hopf HW, Feiner J, Finlay HE, Takahashi M, Bostrom A, Songster C, Aminoff MJ (2005). Acute isovolemic anemia impairs central processing as determined by P300 latency. Clin Neurophysiol.

[35] Nur E, Kim Y-S, Truijen J, van Beers EJ, Davis SCAT, Brandjes DP, Biemond BJ, van Lieshout JJ (2009). Cerebrovascular reserve capacity is impaired in patients with sickle cell disease. Blood.

[36] Silvestrini M, Vernieri F, Pasqualetti P, Matteis M, Passarelli F, Troisi E, Caltagirone C. Impaired cerebral vasoreactivity and risk of stroke in patients with asymptomatic carotid artery stenosis. JAMA. 2000;283(16):2122-7. 10.1001/jama.283.16.2122 10791504

[37] Chipman AM, Jenne C, Wu F, Kozar RA (2020). Contemporary resuscitation of hemorrhagic shock: what will the future hold?. Am J Surg.

[38] Semple JW, Rebetz J, Kapur R (2019). Transfusion-associated circulatory overload and transfusion-related acute lung injury. Blood.

[39] Sekhon MS, McLean N, Henderson WR, Chittock DR, Griesdale DE (2012). Association of hemoglobin concentration and mortality in critically ill patients with severe traumatic brain injury. Crit Care.

[40] Oddo M, Milby A, Chen I, Frangos S, MacMurtrie E, Maloney-Wilensky E, Stiefel M, Kofke WA, Levine JM, Le Roux PD (2009). Hemoglobin concentration and cerebral metabolism in patients with Aneurysmal Subarachnoid Hemorrhage. Stroke.

[41] Alberts MJ, Batjer HH, Shaibani A, Ault ML, Drescher J, Naidech AM (2006). Higher hemoglobin is Associated with less cerebral infarction, poor outcome, and death after subarachnoid hemorrhage. Neurosurgery.

[42] Naidech AM, Jovanovic B, Wartenberg KE, Parra A, Ostapkovich N, Connolly ES, Mayer SA, Commichau C (2007). Higher hemoglobin is associated with improved outcome after subarachnoid hemorrhage*. Crit Care Med.

[43] Wu Y, Lin F, Bai Y, Liang F, Wang X, Wang B, Jian M, Wang Y, Liu H, Wang A, et al. Early stellate ganglion block for improvement of postoperative cerebral blood flow velocity after aneurysmal subarachnoid hemorrhage: results of a pilot randomized controlled trial. J Neurosurg. 2023;139(5):1339-47. 10.3171/2023.3.JNS222567 37119094

[44] Geraghty JR, Lung TJ, Hirsch Y, Katz EA, Cheng T, Saini NS, Pandey DK, Testai FD (2021). Systemic Immune-inflammation index predicts delayed cerebral vasospasm after Aneurysmal Subarachnoid Hemorrhage. Neurosurgery.

[45] Smith MJ, Le Roux PD, Elliott JP, Winn HR (2004). Blood transfusion and increased risk for vasospasm and poor outcome after subarachnoid hemorrhage. J Neurosurg.

[46] Boutin A, Moore L, Lauzier F, Chasse M, English S, Zarychanski R, McIntyre L, Griesdale D, Fergusson DA, Turgeon AF (2017). Transfusion of red blood cells in patients with traumatic brain injuries admitted to Canadian trauma health centres: a multicentre cohort study. BMJ Open.

[47] Boutin A, Moore L, Green RS, Zarychanski R, Erdogan M, Lauzier F, English S, Fergusson DA, Butler M, McIntyre L (2018). Hemoglobin thresholds and red blood cell transfusion in adult patients with moderate or severe traumatic brain injuries: a retrospective cohort study. J Crit Care.

[48] Moman RN, Kor DJ, Chandran A, Hanson AC, Schroeder DR, Rabinstein AA, Warner MA (2019). Red blood cell transfusion in acute brain injury subtypes: an observational cohort study. J Crit Care.

[49] Leal-Noval SR, Munoz-Serrano A, Arellano-Orden V, Cayuela A, Munoz-Gomez M, Recio A, Alcantara A, Amaya-Villar R, Casado-Mendez M, Murillo-Cabezas F (2016). Effects of Red Blood Cell Transfusion on Long-Term disability of patients with traumatic brain Injury. Neurocrit Care.

[50] Vedantam A, Yamal JM, Rubin ML, Robertson CS, Gopinath SP (2016). Progressive hemorrhagic injury after severe traumatic brain injury: effect of hemoglobin transfusion thresholds. J Neurosurg.

