# Supplementary material for: Red blood cell transfusion in neurocritical patients: a systematic review and meta-analysis
Source: BMC Anesthesiol. 2024 Mar 19;24:106. doi: 10.1186/s12871-024-02487-9 (PMC10949741; doi:10.1186/s12871-024-02487-9)
Supplement: Supplementary file 1 — Supplementary Material 1. [file 12871_2024_2487_MOESM1_ESM.docx]

**Red blood cell transfusion in neurocritical patients: A systematic review and meta-analysis**

**Supplemental**

**Table of contents**

[Supplemental Figure 1. Unfavorable GOS outcomes at six months 4](#_Toc158573103)

[Supplemental Figure 2. Long-term unfavorable outcomes 4](#_Toc158573104)

[Supplemental Figure 3. Patients transfused in different transfusion strategies 4](#_Toc158573105)

[Supplemental Figure 4. RBC units per patient in different transfusion strategies 4](#_Toc158573106)

[Supplemental Figure 5. The impact of different transfusion strategies on vasospasm 4](#_Toc158573107)

[Supplemental Figure 6. The impact of different transfusion strategies on stroke 5](#_Toc158573108)

[Supplemental Figure 7. The impact of different transfusion strategies on intracranial hypertension requiring therapy 5](#_Toc158573109)

[Supplemental Figure 8. The impact of different transfusion strategies on DVT 5](#_Toc158573110)

[Supplemental Figure 9. The impact of different transfusion strategies on acute myocardial infarction 5](#_Toc158573111)

[Supplemental Figure 10. The impact of different transfusion strategies on hypotension 5](#_Toc158573112)

[Supplemental Figure 11. The impact of different transfusion strategies on pneumonia 6](#_Toc158573113)

[Supplemental Figure 12. The impact of different transfusion strategies on pulmonary embolus 6](#_Toc158573114)

[Supplemental Figure 13. The impact of different transfusion strategies on ARDS 6](#_Toc158573115)

[Supplemental Figure 14. The impact of different transfusion strategies on urinary tract infection 6](#_Toc158573116)

[Supplemental Table 1. Mortality for neurocritical patients 7](#_Toc158573117)

[Supplemental Table 2. Characteristics of included studies 9](#_Toc158573118)

[Supplemental Table 3. Characteristics of ongoing studies 16](#_Toc158573119)

[Supplemental Digital Content Search strategies 20](#_Toc158573120)

[Legends 23](#_Toc158573121)

# Supplemental Figure 1. Unfavorable GOS outcomes at six months


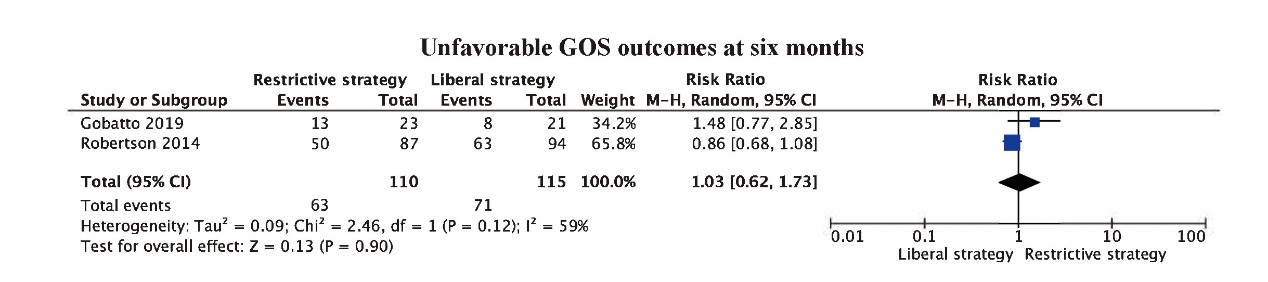


# Supplemental Figure 2. Long-term unfavorable outcomes


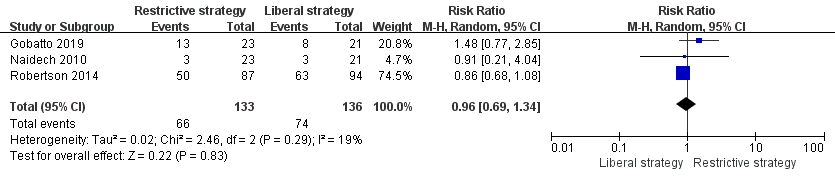


# Supplemental Figure 3. Patients transfused in different transfusion strategies


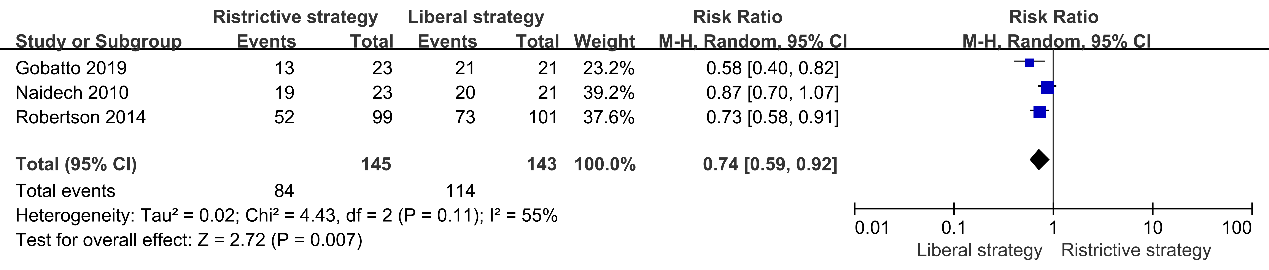


# Supplemental Figure 4. RBC units per patient in different transfusion strategies


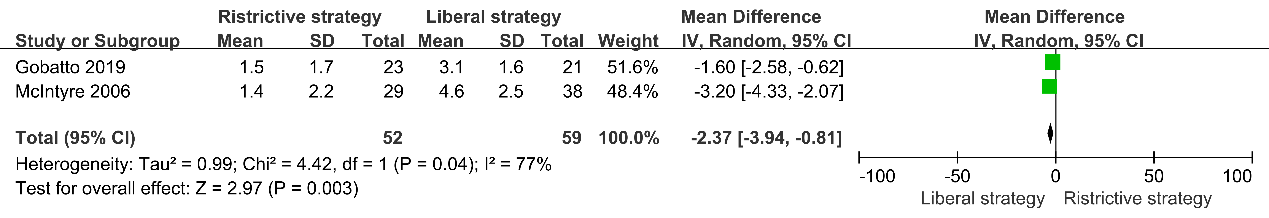


# Supplemental Figure 5. The impact of different transfusion strategies on vasospasm


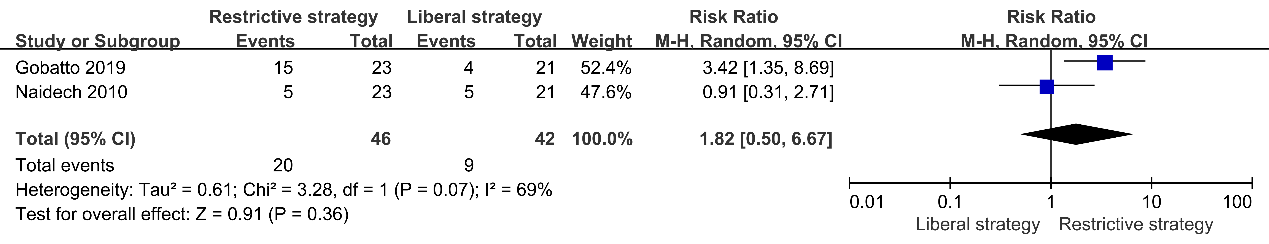


# Supplemental Figure 6. The impact of different transfusion strategies on stroke


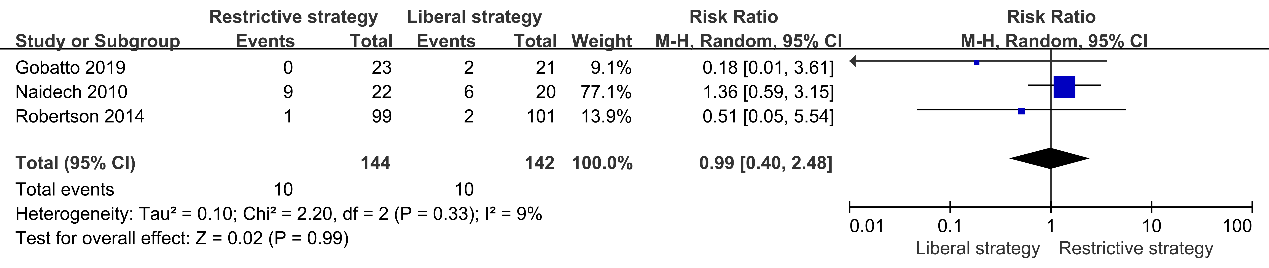


# Supplemental Figure 7. The impact of different transfusion strategies on intracranial hypertension requiring therapy


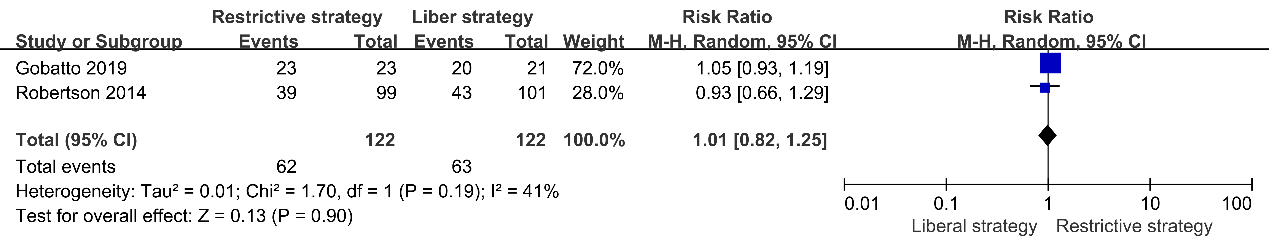


# Supplemental Figure 8. The impact of different transfusion strategies on DVT


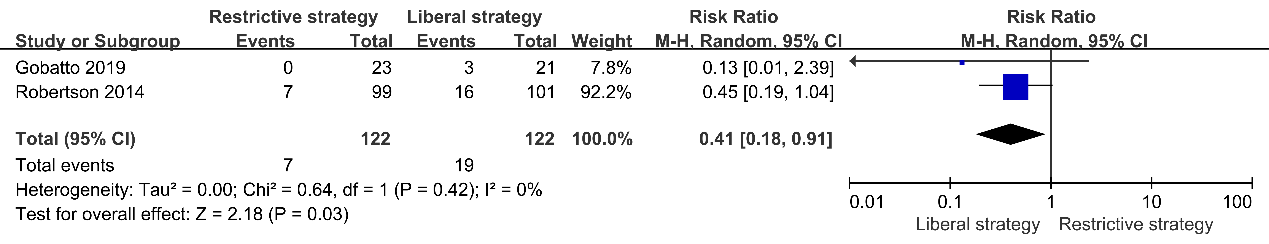


# Supplemental Figure 9. The impact of different transfusion strategies on acute myocardial infarction


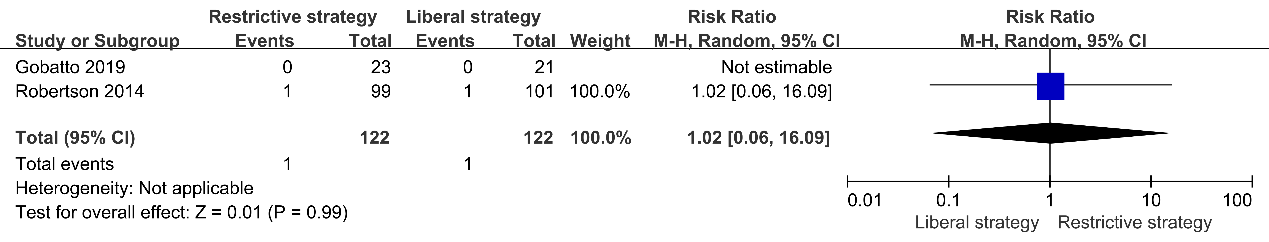


# Supplemental Figure 10. The impact of different transfusion strategies on hypotension


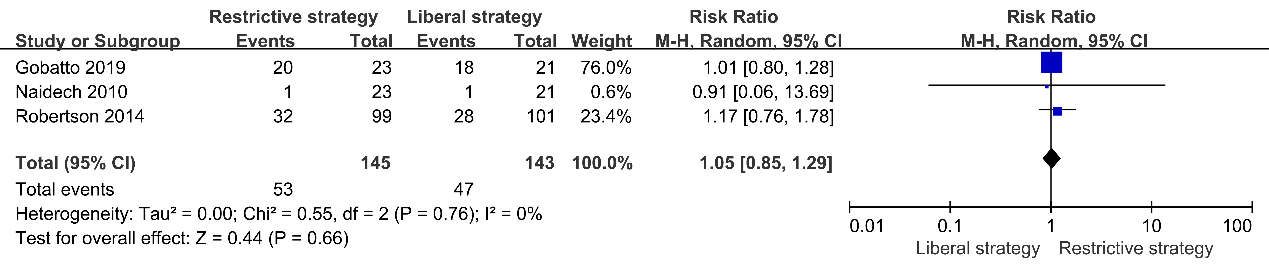


# Supplemental Figure 11. The impact of different transfusion strategies on pneumonia


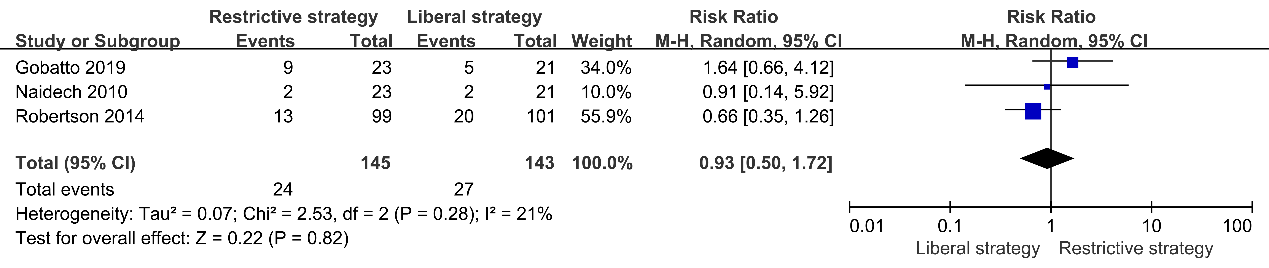


# Supplemental Figure 12. The impact of different transfusion strategies on pulmonary embolus


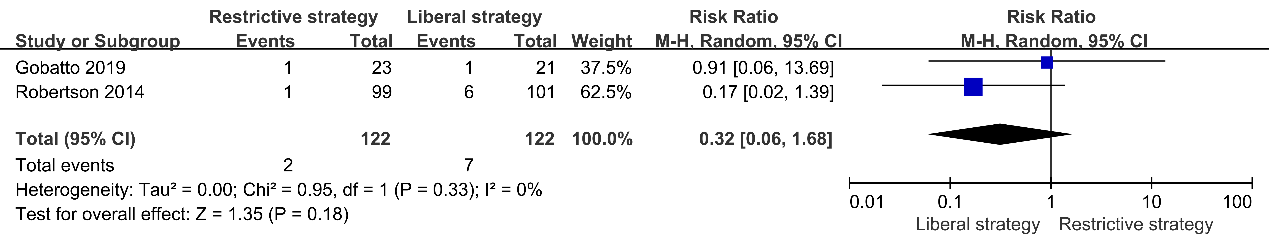


# Supplemental Figure 13. The impact of different transfusion strategies on ARDS


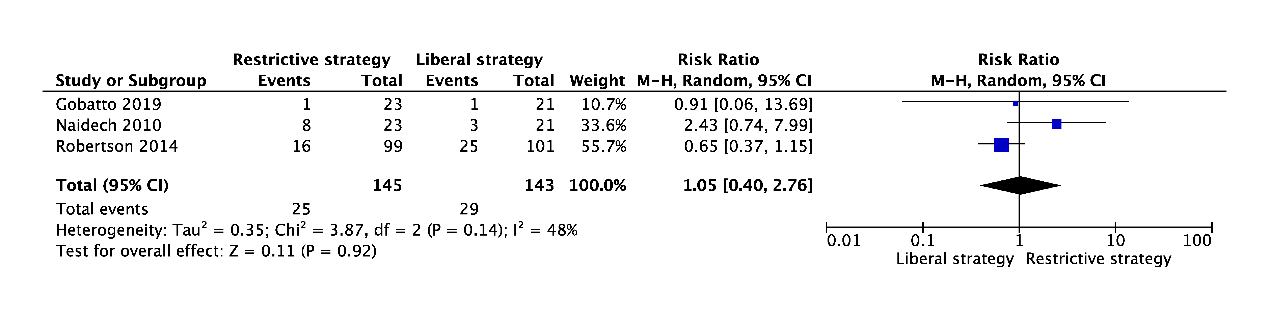


# Supplemental Figure 14. The impact of different transfusion strategies on urinary tract infection


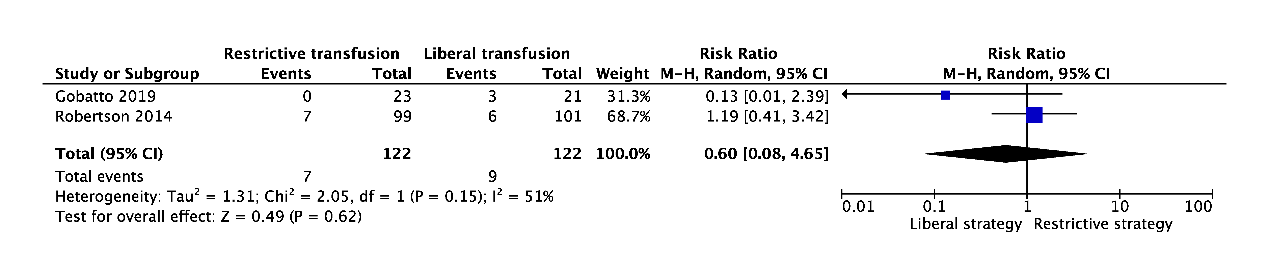


# Supplemental Table 1. Mortality for neurocritical patients

| **Mortality for neurocritical patients** | | | | | | |
| --- | --- | --- | --- | --- | --- | --- |
| **Patient or population:** patients with neurocritical patients **Intervention:** Restrictive transfusion strategy | | | | | | |
| **Outcomes** | **Illustrative comparative risks* (95% CI)** | | **Relative effect (95% CI)** | **No of Participants (studies)** | **Quality of the evidence (GRADE)** | **Comments** |
|  | Assumed risk | Corresponding risk |  |  |  |  |
|  | **Control** | **Mortality** |  |  |  |  |
| **Mortality at 6 months** | **Study population** | | **RR 1.41**  (0.41 to 4.82) | 232 (2 studies) | ⊕⊝⊝⊝ **very low**^1,2,3^ |  |
|  | **161 per 1000** | **227 per 1000** (66 to 776) |  |  |  |  |
|  | **Moderate** | |  |  |  |  |
|  | **135 per 1000** | **190 per 1000** (55 to 651) |  |  |  |  |
| **ICU mortality** | **Study population** | | **RR 2.53**  (0.53 to 12.13) | 111 (2 studies) | ⊕⊕⊝⊝ **low**^4,5^ |  |
|  | **68 per 1000** | **172 per 1000** (36 to 822) |  |  |  |  |
|  | **Moderate** | |  |  |  |  |
|  | **63 per 1000** | **159 per 1000** (33 to 764) |  |  |  |  |
| **In-hospital mortality** | **Study population** | | **RR 2.34**  (0.5 to 11) | 111 (2 studies) | ⊕⊕⊝⊝ **low**^4,5^ |  |
|  | **102 per 1000** | **238 per 1000** (51 to 1000) |  |  |  |  |
|  | **Moderate** | |  |  |  |  |
|  | **90 per 1000** | **211 per 1000** (45 to 990) |  |  |  |  |
| *The basis for the **assumed risk** (e.g. the median control group risk across studies) is provided in footnotes. The **corresponding risk** (and its 95% confidence interval) is based on the assumed risk in the comparison group and the **relative effect** of the intervention (and its 95% CI).  **CI:** Confidence interval; **RR:** Risk ratio; | | | | | | |
| GRADE Working Group grades of evidence **High quality:** Further research is very unlikely to change our confidence in the estimate of effect.  **Moderate quality:** Further research is likely to have an important impact on our confidence in the estimate of effect and may change the estimate. **Low quality:** Further research is very likely to have an important impact on our confidence in the estimate of effect and is likely to change the estimate. **Very low quality:** We are very uncertain about the estimate. | | | | | | |
| ^1^ Robertson 2014 did not mention "Allocation concealment" in the paper. ^2^ I^2^ = 61% ^3^ Gobatto 2019: the sample size is small and the confidence interval is wide ^4^ McIntyre 2006 did not mention "Blinding of outcome assessment" in the paper. ^5^ The sample size is small and the confidence interval is wide. | | | | | | |

# Supplemental Table 2. Characteristics of included studies

| Characteristic | Gobatto 2019 | McIntyre 2006 | Naidech 2010 | Robertson 2014 | Zygun 2009 |
| --- | --- | --- | --- | --- | --- |
| Design | Parallel group RCT | Subgroup of a RCT (TRICC) | Parallel group RCT | randomized trial; factorial (2 × 2) design; Multicenter | Parallel group RCT |
| Location | Brazil | Canada | USA | USA | UK |
| Setting | ICU | ICU | ICU | LevelI trauma centers | Neurosciences Critical Care Unit |
| Sample size | 44 | 67 | 44 | 200 | 30 |
| Recruitment years | 2014-2016 | 1994-1997 | 2006-2008 | 2006-2012 | 2003-2005 |
| Inclusion  criteria | >18 yr; Moderate or severe TBI (GCS score ≤ 12 at hospital admission); Hemoglobin <9 g/dL | Trauma patients within the TRICC trial who had sustained a closed head injury and Hemoglobin ≤ 9.0g/dL within 72 hours of ICU admission | SAH within three days, World Federation of Neurologic Surgeons grades two through four (patients with grade five on admission could be enrolled if they improved to grade four or better) or grade one with thick subarachnoid clot (Columbia CT grading scale 3–4), and aneurysm obliteration imminent or completed | Patients with a closed head injury who were not able to follow commands after resuscitation and could be enrolled in the study within 6 hours of injury | >16 years; severe TBI (GCS score <= 8, intracranial hypertension >20 mmHg for greater than 10 minutes, or requiring neurosurgical intervention) |
| Exclusion  criteria | GCS = 3; Dilated pupils bilaterally; Previous neurological sequelae; Pregnancy; Jehovah's witnesses; Hemorrhagic shock; Moribund | Intensive care, hospital and 90-day mortality rates, ICU and hospital length of stay, and rates of organ failure | patients with cerebral infarction on CT before enrollment | GCS score of 3 with fixed and dilated pupils; penetrating trauma; pregnancy; life-threatening systemic injuries;  severe preexisting disease | Active hemorrhage; active coronary ischemia; inability to place cerebral oxygenation monitors; failure to fall below allocated transfusion threshold during intracranial pressure, and brain tissue oxygen monitoring and lack of informed assent from next of kin |
| Intervention | Transfused if hemoglobin < 7 g/dL | Transfuse one red cell unit when hemoglobin < 7.0 g/dL; Maintain hemoglobin 7.0-9.0 g/dL. | Goal HGB of at least 10 g/dl:  If the HGB was 0.1–1 g/dl below goal, then one unit PRBC was given; if >1 g/dl below goal, then two units were given with follow-up after the transfusion. | Hemoglobin transfusion threshold of 7 g/dL. During the acute postinjury recovery period, the assigned hemoglobin threshold was maintained with transfusion of leukoreduced-packed red blood cells. In patients who were actively bleeding, hemodynamic instability was also used as an indication for transfusion in both transfusion thresholds. | Patients were randomized to one of three transfusion thresholds: 8, 9, or 10 g/dL.  When the patients’ hemoglobin concentration fell below their assigned threshold, two units of packed red blood cells were transfused, each over 1 hour regardless of assigned transfusion threshold. |
| Control | Transfused if hemoglobin < 9 g/dL | Transfused if hemoglobin < 10.0 g/dL; Maintain hemoglobin 10.0-12.0 g/dL | Goal HGB of at least 11.5 g/dl: If the HGB was 0.1–1 g/dl below goal, then one unit PRBC was given; if >1 g/dl below goal, then two units were given with follow-up after the transfusion. | Hemoglobin transfusion threshold of 10 g/dL | Patients were randomized to one of three transfusion thresholds: 8, 9, or 10 g/dL, but the control group was not clarified. |
| Primary outcome | Mean hemoglobin concentration during the 14 days after hospital admission | 30-day all-cause mortality | The primary safety endpoints: the days with a core temperature of at least 100.4F; the number of ventilator-free days (within 13 days of SAH). | GOS at 6 months after the injury; the primary safety outcomes for the transfusion threshold: mortality, the incidence of ARDS, and the incidence of infections (total number of incidences of pneumonia, bacteremia, urinary tract infection, and ventriculitis) | Change in brain tissue oxygen (PbtO_2_) |
| Secondary outcomes | The number of transfused patients; the number of RBC packs transfused; ICU mortality; hospital mortality; mortality at 6 months after hospital discharge; adverse events; presence of elevated ICP and intensity of intracranial hypertension treatment; cerebral hemodynamic findings on sequential TCD analysis; lengths of ICU and hospital stay; ICU-free days; duration of mechanical ventilation; mechanical-ventilation-free days and neurological status (GOS) at hospital discharge and 6 months after hospital discharge. | ICU; hospital and 90-day mortality; ICU and hospital length of stay; rates of organ failure. | Vasospasm; Pneumonia; The safety endpoints: new core temperature of at least 100.4F, symptomatic respiratory distress or radiographic pulmonary edema, hypotension, transaminases at least twice the upper limit of normal, or blood-borne infection; Hgb values; NIH Stroke Scale at 14 days and the mRS at 14 days, 28 days, and 3 months with a validated questionnaire; cerebral infarction; delayed cerebral ischemia | The secondary transfusion threshold outcome: Disability Rating Scale; The secondary outcome was mortality for patients assigned to erythropoietin or placebo. | Dependence of baseline hemoglobin concentration and baseline PbtO_2_ on the relationship of transfusion and PbtO_2_; the effect of transfusion on LPR and brain pH as markers of cerebral metabolic state |
| Length of  follow-up | 6 months | 90 days | 3 months | 6 months | 6 months |
| Trial registration | NCT02203292 | Not mentioned | registered at www.stroketrials.org | NCT00313716 | ISRCTN89085577 |
| Funding source | None | The TRICC study was supported by the Medical Research Council of Canada and by an unrestricted grant from Bayer. | This study was funded by grants to AMN from the Neurocritical Care Society, supported by Novo-Nordisk (for partial salary support) and from the Northwestern Memorial Foundation for MRI scans and additional PRBC transfusions above usual care. | This study was supported by grant P01-NS38660 from the National Institute of Neurological Disorders and Stroke. | G9439390 ID 56833 from Association of Anesthetists of Great Britain and Ireland, Intensive Care Society and Acute Brain Injury Program in the Wolfson Brain Imaging Centre, which was funded by the MRC; the Detweiler Traveling Fellowship of the Royal College of Physicians and Surgeons of Canada, the Meredith Graduate Master’s Scholarship, University of Calgary, the Worker’s Compensation Board of Alberta (DAZ); the Academy of Medical Sciences/The Health Foundation–Senior Surgical Scientist Fellowship (PJH); British Journal of Anesthesia/Royal College of Anesthetists Research Fellowship (JN); Codman (Johnson & Johnson), the Evelyn Trust and a BP-TNK Kapitza scholarship (IT) |

GOS: Glasgow Outcome Scale; ICP: intracranial pressure; ICU: intensive care unit; LPR: lactate pyruvate ratio; PbtO_2_: brain tissue oxygen; TCD: transcranial Doppler ultrasound; TRICC: Transfusion Requirements in Critical Care

# Supplemental Table 3. Characteristics of ongoing studies

| Characteristic | HEMOTION | SAHaRA | TRAIN |
| --- | --- | --- | --- |
| Design | Parallel group RCT  Masking: Single (Outcomes Assessor)  Multicenter | Parallel group RCT  Masking: Single (Outcomes Assessor)  Multicenter | Parallel group RCT  Masking: None (Open Label) |
| Sponsor | CHU de Quebec-Universite Laval | Ottawa Hospital Research Institute | Erasme University Hospital |
| Location | Brazil, Canada, France, UK | Australia, Canada, United States | Belgium |
| Sample size | 742 | 740 | 794 |
| Recruitment Status | Recruiting | Recruiting | Recruiting |
| Actual Study Start Date | September 1, 2017 | February 12, 2018 | September 13, 2016 |
| Estimated Study Completion | January 1, 2024 | September 2024 | July 30, 2023 |
| Inclusion  criteria | >18 yr; Acute moderate to severe blunt TBI; GCS ≤ 12; Hb level ≤ 100 g/L | ≥18 yr; First ever episode of aneurysmal SAH (aSAH); Hb ≤100g/L within 10 days following aSAH | 18-80 yrs; GCS ≤ 13 on randomization; Expected ICU stay > 72 hours; Hb ≤ 9 g/dL within 10 days from brain injury |
| Exclusion  criteria | Received transfusion after ICU admission; Contraindications or objection to transfusions; GCS of 3 with dilated fixed pupils; Brain dead; Active life-threatening bleeding with hemorrhagic shock or requiring urgent surgical procedure; A decision to withhold or withdraw life-sustaining therapies was made; No fixed address | Physician and or family decision to withdraw/withhold active medical care; Active bleeding with hemodynamic instability; Patients with contraindication or known objection to blood transfusions  SAH due to mycotic aneurysm, infundibulum and vascular malformations | Post-anoxic coma; status epilepticus without underlying brain injury; CNS infections; Known previous neurological disease, causing significant cognitive and/or motor handicap; ICH due to AVM or brain tumor; Inability or reduced ability to receive blood products; Active and uncontrolled bleeding; GCS of 3 with both pupils fixed and dilated; brain death or imminent death; Pregnancy; Medical need to correct anemia with target Hb levels > 9 g/dL; do-not-escalate orders; Previous allo-immunization due to transfusion |
| Intervention | Patients will receive red blood cells transfusion if Hb ≤ 100 g/L | Liberal RBC Transfusion Strategy: Hemoglobin value of ≤100g/L | Liberal Transfusion Strategy: "Blood Transfusion" will be given when hemoglobin concentration will be below 9 g/dL |
| Control | Patients will receive red blood cells transfusion if Hb ≤ 70 g/L | Restrictive RBC Transfusion Strategy: Hemoglobin value of ≤80g/L | Restrictive Transfusion Strategy: "Blood Transfusion" will be given when hemoglobin concentration will be below 7 g/dL |
| Primary outcome | GOSe at 6 months | mRS at 12 months | Unfavorable Neurological Outcome (defined by the GOSe of 1-5) at 180 days |
| Secondary Outcome | Mortality at ICU, Hospital and at 6 months; FIM at 6 months; Quality of life (EQ-5D) at 6 months; Quality of life (Qolibri questionnaires) at 6 months;  Depression (PHQ-9) at 6 months; Complications related to transfusion at 6 months | FIM at 12 months  EuroQOL Quality of Life Scale (EQ5D) at 12 months;  Red Blood Cell Transfusions within 21 days; Daily Hemoglobin within 21 days; Transfusion-related Complications within 28 days; Delayed Cerebral Ischemia and Vasospasm within 28 days; Cerebral Infarction within 28 days; Mechanical Ventilation within 21 days; Length of Stay; Mortality at 12 months | Survival at 28 days; Changes in the Glasgow Coma Score (GCS) over time at 28 days; ICU length of stay; Hospital length of stay; Presence and severity of extra-cerebral organ dysfunction/failure at 28 days; Infection rate at 28 days; Composite outcome at 28 days; Brain Oxygen Pressure at 28 days; Daily Fluid Balance at 28 days; SAE at 28 days |
| Length of  follow-up | 6 months | 12 months | 6 months |
| Trial registration | NCT03260478 | NCT03309579 | NCT02968654 |
| Protocol published | Yes | Yes | Yes |

AVM: arterio-venous malformation; CNS: central nervous system; FIM: Functional Independence Measure; GCS: Glasgow Coma Scale; GOSe: extended Glasgow Outcome Scale; Hb: hemoglobin; mRS: Modified Rankin Scale; SAE: Serious Adverse Events.

# Supplemental Digital Content Search strategies

**Search strategies of CENTRAL**

#1 MeSH descriptor: [Erythrocyte Transfusion] explode all trees

#2 MeSH descriptor: [Blood Transfusion] explode all trees

#3 MeSH descriptor: [Blood Component Transfusion] explode all trees

#4 MeSH descriptor: [Hemoglobins] explode all trees

#5 ((Erythrocyte Transfusion) OR (Red Blood Cell Transfusion) OR (Blood Transfusion) OR (Blood Component Transfusion) OR Hemoglobin OR Eryhem OR (Ferrous Hemoglobin)):ti,ab,kw

#6 MeSH descriptor: [Anemia] explode all trees

#7 Anemia:ti,ab,kw

#8 #1 OR #2 OR #3 OR #4 OR #5 OR #6 OR #7

#9 MeSH descriptor: [Cerebral Hemorrhage] explode all trees

#10 ((Cerebral Parenchymal Hemorrhage) OR (Intracerebral Hemorrhage) OR (Cerebral Hemorrhage) OR (Brain Hemorrhage)):ti,ab,kw

#11 MeSH descriptor: [Intracranial Hemorrhage, Hypertensive] explode all trees

#12 ((hypertensive intracranial hemorrhage) OR (hypertensive cerebral hemorrhage) OR (hypertensive intracerebral hemorrhage) OR (subarachnoid hemorrhage)):ti,ab,kw

#13 MeSH descriptor: [Cerebral Hemorrhage, Traumatic] explode all trees

#14 ((Traumatic Cerebral Hemorrhage) OR (Traumatic Cerebral Parenchymal Hemorrhage) OR (Traumatic Intracerebral Hemorrhage) OR (Traumatic Cerebral Intraparenchymal Hemorrhage) OR (Traumatic Cerebral Hematoma) OR (Traumatic Cerebral Intraparenchymal Hematoma)):ti,ab,kw

#15 MeSH descriptor: [Hematoma, Epidural, Cranial] explode all trees

#16 ((Cranial Epidural Hemorrhage) OR (Cranial Extradural Hematoma) OR (Intracranial Epidural Hematoma) OR (Cranial Epidural Hematoma) OR (Cranial Extradural Hemorrhage)):ti,ab,kw

#17 MeSH descriptor: [Hematoma, Subdural, Intracranial] explode all trees

#18 (Intracranial Subdural Hematoma):ti,ab,kw

#19 MeSH descriptor: [Brain Injuries, Traumatic] explode all trees

#20 ((Traumatic Brain Injuries) OR (Brain Trauma) OR (Brain Traumas) OR (Traumatic Brain Injury) OR (Traumatic Encephalopathies) OR (Traumatic Encephalopathy) OR (head injury) OR (head trauma) OR (decompressive craniectomy)):ti,ab,kw

#21 #9 OR #10 OR #11 OR #12 OR #13 OR #14 OR #15 OR #16 OR #17 OR #18 OR #19 OR #20

#22 #8 AND #21

**Search strategies of Embase**

#1 ‘randomized controlled trial’/exp

#2 ‘controlled clinical trial’/exp

#3 randomized:ab,ti

#4 randomised:ab,ti

#5 placebo:ab,ti

#6 randomly:ab,ti

#7 trial:ab,ti

#8 groups:ab,ti

#9 #1 OR #2 OR #3 OR #4 OR #5 OR #6 OR #7 OR #8

#10 'animals'/exp NOT 'humans'/exp

#11 #9 NOT #10

#12 'erythrocyte transfusion'/exp

#13 'blood transfusion'/exp

#14 'blood component therapy'/exp

#15 'hemoglobin'/exp

#16 'anemia'/exp

#17 anemia:ab,ti

#18 ((Erythrocyte Transfusion) OR (Red Blood Cell Transfusion) OR (Blood Transfusion) OR (Blood Component Transfusion) OR Hemoglobin OR Eryhem OR (Ferrous Hemoglobin)):ab,ti

#19 #12 OR #13 OR #14 #15 OR #16 OR #17 OR #18

#20 'brain hemorrhage'/exp

#21 ((Cerebral Parenchymal Hemorrhage) OR (Intracerebral Hemorrhage) OR (Cerebral Hemorrhage) OR (Brain Hemorrhage)):ab,ti

#22 'subarachnoid hemorrhage'/exp

#23 ((hypertensive intracranial hemorrhage) OR (hypertensive cerebral hemorrhage) OR (hypertensive intracerebral hemorrhage) OR (subarachnoid hemorrhage)):ab,ti

#24 ‘epidural hematoma’/exp

#25 ((Cranial Epidural Hemorrhage) OR (Cranial Extradural Hematoma) OR (Intracranial Epidural Hematoma) OR (Cranial Epidural Hematoma) OR (Cranial Extradural Hemorrhage)):ab,ti

#26 ‘subdural hematoma’/exp

#27 (Intracranial Subdural Hematoma):ab,ti

#28 ‘traumatic brain injury’/exp

#29 ((Traumatic Brain Injuries) OR (Brain Trauma) OR (Brain Traumas) OR (Traumatic Brain Injury) OR (Traumatic Encephalopathies) OR (Traumatic Encephalopathy) OR (head injury) OR (head trauma) OR (decompressive craniectomy)):ab,ti

#30 #20 OR #21 OR #22 OR #23 OR #24 OR #25 OR #26 OR #27 OR #28 OR #29

#31 #11 AND #19 AND #30

**Search strategies of PubMed**

#1 randomized controlled trial [pt]

#2 controlled clinical trial [pt]

#3 randomized [tiab]

#4 placebo [tiab]

#5 drug therapy [sh]

#6 randomly [tiab]

#7 trial [tiab]

#8 groups [tiab]

#9 #1 OR #2 OR #3 OR #4 OR #5 OR #6 OR #7 OR #8

#10 animals [mh] NOT humans [mh]

#11 #9 NOT #10

#12 Erythrocyte Transfusion [mh]

#13 Blood Transfusion [mh]

#14 Blood Component Transfusion [mh]

#15 Hemoglobins [mh]

#16 Erythrocyte Transfusion OR Red Blood Cell Transfusion OR Blood Transfusion OR Blood Component Transfusion OR Hemoglobin OR Eryhem OR Ferrous Hemoglobin [tiab]

#17 Anemia [mh]

#18 Anemia [tiab]

#19 #12 OR #13 OR #14 OR #15 OR #16 OR #17 OR #18

#20 Cerebral Hemorrhage [mh]

#21 Cerebral Parenchymal Hemorrhage OR Intracerebral Hemorrhage OR Cerebral Hemorrhage OR Brain Hemorrhage [tiab]

#22 Intracranial Hemorrhage, Hypertensive [mh]

#23 hypertensive intracranial hemorrhage OR hypertensive cerebral hemorrhage OR hypertensive intracerebral hemorrhage OR subarachnoid hemorrhage [tiab]

#24 Cerebral Hemorrhage, Traumatic [mh]

#25 Traumatic Cerebral Hemorrhage OR Traumatic Cerebral Parenchymal Hemorrhage OR Traumatic Intracerebral Hemorrhage OR Traumatic Cerebral Intraparenchymal Hemorrhage OR Traumatic Cerebral Hematoma OR Traumatic Cerebral Intraparenchymal Hematoma [tiab]

#26 Hematoma, Epidural, Cranial [mh]

#27 Cranial Epidural Hemorrhage OR Cranial Extradural Hematoma OR Intracranial Epidural Hematoma OR Cranial Epidural Hematoma OR Cranial Extradural Hemorrhage [tiab]

#28 Hematoma, Subdural, Intracranial [mh]

#29 Intracranial Subdural Hematoma [tiab]

#30 Brain Injuries, Traumatic [mh]

#31 Traumatic Brain Injuries OR Brain Trauma OR Brain Traumas OR Traumatic Brain Injury OR Traumatic Encephalopathies OR Traumatic Encephalopathy OR head injury OR head trauma OR decompressive craniectomy [tiab]

#32 #20 OR #21 OR #22 OR # 23 OR #24 OR #25 OR #26 OR #27 OR #28 OR #29 OR #30 OR #31

#33 #11 AND #19 AND #32

# Legends

Supplemental Figure 1. Unfavorable GOS outcomes at six months

Supplemental Figure 2. Long-term unfavorable outcomes

Supplemental Figure 3. Patients transfused in different transfusion strategies

Supplemental Figure 4. RBC units per patient in different transfusion strategies

RBC, Red blood cell

Supplemental Figure 5. The impact of different transfusion strategies on vasospasm

Supplemental Figure 6. The impact of different transfusion strategies on stroke

Supplemental Figure 7. The impact of different transfusion strategies on intracranial hypertension requiring therapy

Supplemental Figure 8. The impact of different transfusion strategies on DVT

DVT, Deep venous thrombosis

Supplemental Figure 9. The impact of different transfusion strategies on acute myocardial infarction

Supplemental Figure 10. The impact of different transfusion strategies on hypotension

Supplemental Figure 11. The impact of different transfusion strategies on pneumonia

Supplemental Figure 12. The impact of different transfusion strategies on pulmonary embolus

Supplemental Figure 13. The impact of different transfusion strategies on ARDS

ARDS, Acute respiratory distress syndrome

Supplemental Figure 14. The impact of different transfusion strategies on urinary tract infection

Supplemental Table 1. Characteristics of included studies

GOS, Glasgow Outcome Scale; ICP, Intracranial pressure; ICU, Intensive care unit; LPR, Lactate pyruvate ratio; PbtO2, Brain tissue oxygen; TCD, Transcranial doppler ultrasound; TRICC, Transfusion requirements in critical care

Supplemental Table 2. Characteristics of ongoing studies

AVM, Arterio-venous malformation; CNS, Central nervous system; FIM, Functional independence measure; GCS, Glasgow coma scale; GOSe, Extended glasgow outcome scale; Hb, Hemoglobin; mRS, Modified rankin scale; SAE, Serious adverse events.
